# A prospective study of androgen levels, hormone-related genes and risk of rheumatoid arthritis

**DOI:** 10.1186/ar2742

**Published:** 2009-06-25

**Authors:** Elizabeth W Karlson, Lori B Chibnik, Monica McGrath, Shun-Chiao Chang, Brendan T Keenan, Karen H Costenbader, Patricia A Fraser, Shelley Tworoger, Susan E Hankinson, I-Min Lee, Julie Buring, Immaculata De Vivo

**Affiliations:** 1 Division of Rheumatology, Immunology, and Allergy, Department of Medicine, Brigham and Women's Hospital and Harvard Medical School, 75 Francis Street, Boston, MA 02115, USA; 2 Channing Laboratory, Department of Medicine, Brigham and Women's Hospital and Harvard Medical School, 75 Francis Street, Boston, MA 02115, USA; 3 Department of Epidemiology, Harvard School of Public Health, 677 Huntington Avenue, Boston, MA 02115, USA; 4 Immune Disease Institute, 800 Huntington Avenue, Boston, MA 02115, USA; 5 Genzyme Corporation, 500 Kendall Street, Cambridge, MA 02115, USA; 6 Division of Preventive Medicine, Department of Medicine, Brigham and Women's Hospital and Harvard Medical School, 75 Francis Street, Boston, MA 02115, USA; 7 Division of Aging, Department of Medicine, Brigham and Women's Hospital and Harvard Medical School, 75 Francis Street, Boston, MA 02115, USA; 8 Department of Ambulatory Care and Prevention, Harvard Medical School, 25 Shattuck Street, Boston, MA 02115, USA

## Abstract

**Introduction:**

Rheumatoid arthritis (RA) is more common in females than males and sex steroid hormones may in part explain this difference. We conducted a case–control study nested within two prospective studies to determine the associations between plasma steroid hormones measured prior to RA onset and polymorphisms in the androgen receptor (*AR*), estrogen receptor 2 (*ESR2*), aromatase (*CYP19*) and progesterone receptor (*PGR*) genes and RA risk.

**Methods:**

We genotyped *AR*, *ESR2*, *CYP19*, *PGR *SNPs and the *AR *CAG repeat in RA case–control studies nested within the Nurses' Health Study (NHS), NHS II (449 RA cases, 449 controls) and the Women's Health Study (72 cases, and 202 controls). All controls were matched on cohort, age, Caucasian race, menopausal status, and postmenopausal hormone use. We measured plasma dehydroepiandrosterone sulfate (DHEAS), testosterone, and sex hormone binding globulin in 132 pre-RA samples and 396 matched controls in the NHS cohorts. We used conditional logistic regression models adjusted for potential confounders to assess RA risk.

**Results:**

Mean age of RA diagnosis was 55 years in both cohorts; 58% of cases were rheumatoid factor positive at diagnosis. There was no significant association between plasma DHEAS, total testosterone, or calculated free testosterone and risk of future RA. There was no association between individual variants or haplotypes in any of the genes and RA or seropositive RA, nor any association for the *AR *CAG repeat.

**Conclusions:**

Steroid hormone levels measured at a single time point prior to RA onset were not associated with RA risk in this study. Our findings do not suggest that androgens or the *AR*, *ESR2*, *PGR*, and *CYP19 *genes are important to RA risk in women.

## Introduction

Women are two to four times more likely than men to develop rheumatoid arthritis (RA) [1,2], and sex hormones including androgens, estrogen, and progesterone may be related to this disparity [3,4]. In women and men the age-related increased incidence of RA parallels the decline in androgen production [5]. Cross-sectional studies of serum androgen levels demonstrate low serum testosterone levels and dehydroepiandrosterone sulfate (DHEAS) in RA patients compared with healthy individuals [6-10]. Serum testosterone levels are inversely correlated with RA disease activity [11], and DHEAS levels are inversely correlated with both disease duration and clinical severity of RA [12]. Androgen receptor expression is significantly higher in RA synovial tissue compared with that in noninflamed synovial tissue [13]. In synovial fluid from active RA patients compared with control individuals, there is evidence of higher free estrogen, lower free androgen levels, and locally elevated aromatase activity [14]. Small randomized controlled trials of testosterone treatment demonstrate significantly improved RA symptoms in women with RA [15] and in men with RA [16]. Whether low androgen levels precede the onset of RA or are simply the result of the disease or its treatment is not clear. One small prospective study demonstrated low DHEAS among premenopausal pre-RA women compared with control individuals [17], while another study demonstrated no differences in total testosterone or DHEAS levels in male and female pre-RA cases compared with sex-matched control individuals [18].

Androgens have immunosuppressive effects on both the humoral and cellular immune response [19-24]. The female sex predominance in RA may be related to low androgen levels prior to disease onset since adrenal and gonadal androgen deficiency can trigger inflammatory cytokines such as TNFα and IL-6, key cytokines responsible for the inflammatory response in RA [25]. Alternatively, androgens may influence RA risk indirectly through conversion to estradiol by aromatase or directly by binding to the androgen receptor and affecting cell proliferation. We hypothesized that low total and free testosterone levels and low DHEAS levels measured before the onset of disease would be associated with an increased risk of RA in women.

Excess estrogen and progesterone may have a protective role in RA etiology. Women are at decreased risk of developing RA during pregnancy, when estrogen and progesterone levels are high. The 12-month postpartum period, particularly the first 3 months, represents a period of increased risk, however, when estrogen and progesterone levels fall dramatically [26]. Progesterone, as well as estrogen and androgens, may therefore play a role in RA pathogenesis.

Based on the hypothesis that a low androgen–estrogen balance is associated with RA in women [3,4], we investigated a number of hormone receptor genes involved in androgen–estrogen pathways for association with RA. The estrogen receptor, the androgen receptor and the progesterone receptor are members of the nuclear receptor superfamily, which depend on ligand binding for activation.

The androgen receptor gene (*AR*) located on chromosome X encodes the androgen receptor, and upon androgen binding the activated androgen–androgen receptor complex activates the expression of other genes via ligand binding, homodimerization, nuclear translocation, DNA binding, and formation of complexes with co-activators and co-repressors [27]. Exon 1 contains a polymorphic CAG repeat sequence that correlates inversely with *AR *transactivational activity [28,29]. Shorter CAG repeat polymorphisms (more active receptor) in *AR *are associated with higher serum androgen levels among premenopausal women [30].

When androgens are converted to their corresponding estrogens, the effects are mediated by estrogen receptors 1 and 2. The estrogen receptor 2 gene (*ESR2*) is located on chromosome 14q22-24. Estrogen receptors are highly expressed on synovial cells [13] and are found on T lymphocytes [31].

The progesterone gene (*PGR*) is a single-copy gene located on chromosome 11q22-23 [32] and has two identified isoforms, *PGR-A *and *PGR-B *[33,34]. Progesterone downregulates the production of the inflammatory chemokine IL-8 at the transcriptional level [35]. The polymorphism (+331G/A), identified by our group [36], creates a novel transcription start site that increases transcriptional activity and alters the *PGR *isoform ratio. The anti-inflammatory role of the progesterone receptor is mediated by *PGR-A*; however, in the presence of the variant (isoform A) there is overproduction of the *PGR-B *isoform [36].

The aromatase gene (*CYP19*) encodes aromatase, which catalyzes the aromatization of the androgens androstenedione and testosterone to estrone and estradiol, respectively. Aromatase has been found in synoviocytes [37]. Data from Cutolo and colleagues suggest an accelerated peripheral metabolic conversion of upstream androgen precursors to 17β-estradiol occurs in RA [3], perhaps via inflammatory cytokines that markedly stimulate aromatase activity in peripheral tissues [38,39]. Moreover, genetic variants in *CYP19 *have been shown to influence endogenous estrogen levels [40].

The overall goal of the present study is to define the contribution of sex-steroid hormone levels measured in plasma samples collected prior to the onset of RA, and the role of genetic variants in hormones in the steroid pathway in RA etiology. We aimed specifically to assess the association between plasma hormone levels for total testosterone, free testosterone, and DHEAS, as well as genetic polymorphisms in the androgen receptor (*AR*), estrogen receptor 2 (*ESR2*), progesterone receptor (*PGR*), *CYP19 *and risk of RA in women. The study pools data and analysis of prospective collected blood samples from several large female cohorts, the Nurses' Health Study (NHS), the NHS II, and the Women's Health Study (WHS).

## Materials and methods

### Study population

The NHS is a prospective cohort of 121,700 female nurses, aged 30 to 55 years in 1976. From 1989 to 1990, 32,826 (27%) NHS participants aged 43 to 70 years provided blood samples for future studies. Further, among women who did not give blood in 1989 to 1990, 33,040 provided buccal cell samples (27% of NHS) for a total of 65,866 DNA samples (54% of the cohort).

The NHS II is a similar prospective cohort, established in 1989, with 116,609 female nurses aged 25 to 42 years. Between 1996 and 1999, 29,611 (25%) NHS II cohort members, aged 32 to 52 at that time, also agreed to provide blood samples for future studies. For the NHS cohorts, blood samples were collected in heparinized tubes and were sent by overnight courier in chilled containers.

The WHS was a randomized, double-blind, placebo-controlled trial designed to evaluate the benefits and risks of low-dose aspirin and vitamin E in the primary prevention of cardiovascular disease and cancer among 39,876 female health professionals, aged 45 years and older, conducted between 1992 and 2004 [41-43]. Following the end of the trial, women who were willing to continue participated in an observational follow-up study. From 1992 through 1995, 28,345 women in the WHS provided blood samples in ethylenediamine tetraacetic acid tubes. On receipt, the blood samples were centrifuged, aliquoted into plasma, red blood cells, and buffy coat fractions, and stored in the vapor phase of liquid nitrogen freezers since collection.

All women in these cohorts completed an initial questionnaire regarding diseases, lifestyle, and health practices, and have been followed biennially in the NHS cohorts and annually in the WHS cohort by questionnaire to update exposures and disease diagnoses. All subjects provided informed consent. All aspects of this study were approved by the Partners' HealthCare Institutional Review Board.

### Identification of rheumatoid arthritis

As previously described [44], we confirmed self-reports of RA based on the presence of RA symptoms on a connective tissue disease screening questionnaire [45] and based on medical record review for American College of Rheumatology (ACR) classification criteria for RA [46]. Subjects with four of the seven ACR criteria documented in the medical record were considered to have definite RA. For this nested case–control study, we also included a small number of subjects (n = 14) with agreement by two rheumatologists on the diagnosis of RA who had three documented ACR criteria for RA and a diagnosis of RA by their physician.

### Population for analysis

For both cases and controls, we excluded women who reported any cancer (except nonmelanoma skin cancer) at baseline or during follow-up, as cancer and its treatment can affect biomarker levels. In the NHS/NHS II, each case with confirmed incident or prevalent RA with buccal samples was matched on year of birth, race/ethnicity, menopausal status, and postmenopausal hormone use to a single healthy woman in the same cohort without RA. In the WHS, we matched each case to three controls on the same factors. For plasma hormone assays and DNA from buffy coat samples, three controls for each confirmed incident RA case in the NHS cohorts were randomly chosen from subjects with stored blood, matching on the same factors plus time of day and fasting status at blood draw. For premenopausal women in NHS II, we also matched on timing of blood sample in the menstrual cycle. To minimize potential population stratification, we limited the analyses to Caucasians for genetic analyses.

### Laboratory assays

The laboratories selected for this study had high assay precision. The laboratories underwent rigorous pilot testing with blinded aliquots from NHS specimens. The laboratory staff were blinded to the case–control status in study samples. Samples were labeled by number only, and matched case–control pairs were handled together identically, shipped in the same batch, and assayed in the same run. The order within each case–control pair was random. Aliquots from pooled quality-control specimens, indistinguishable from study specimens, were interspersed randomly among case–control samples to monitor quality control.

Total testosterone was assayed by specific radioimmunoassay with a solvent extraction step before celite column chromatography [47] at Quest Laboratory, San Juan Capistrano, California. Performance of this assay at Quest Laboratory has been extensively tested in prior NHS study samples with hormone stability studies, test–retest studies, testing duplicate samples, and embedding samples with known values within studies. DHEAS was measured by radioimmunoassay (Diagnostic Systems Laboratories, Webster, TX, USA) at Children's Hospital, Rifai Laboratory (Boston, MA, USA). Sex hormone binding globulin (SHBG) was assayed using a fully automated system (Immulite; DPC, Inc., Los Angeles, CA, USA) at the Reproductive Endocrinology Laboratory at Massachusetts General Hospital, using a solid-phase two-site chemiluminescent enzyme immunometric assay. SHBG levels were used to calculate free testosterone levels [48].

The interassay coefficient of variations for quality-control samples were 14% for testosterone, 13% for SHBG, and 4% for DHEAS. Hormone assays were performed in the NHS cohorts but were not performed in the WHS cohort due to nonsignificant findings in the NHS.

### DNA extraction

DNA was extracted from buffy coats or from buccal cell samples (collected by mouthwash swish and spit procedures) and processed via the QIAmp™ (QIAGEN Inc., Chatsworth, CA, USA) 96-spin blood kit protocol. All genomic DNA samples had an aliquot put through a whole-genome amplification protocol using the GenomPhi DNA amplification kit (GE Healthcare, Piscataway, NJ, USA) to yield high-quality DNA sufficient for SNP genotyping.

### SNP genotyping

DNA was genotyped using Taqman SNP allelic discrimination on the ABI 7900HT using published primers. We used data from the National Cancer Institute Breast and Prostate Cancer Cohort consortium or from the Multi-Ethnic Cohort to select haplotype tagging SNPs for our study [49-51]. SNPs were selected based on resequencing the coding exons of *AR*, *ESR2*, and *CYP19 *in a panel of 95 women from the Multi-Ethnic Cohort (19 each from African Americans, Latinos, Japanese, Americans, Native Hawaiians and Whites), with invasive, non-localized breast cancer. SNPs with minor allele frequency >5% overall or >1% in any one ethnic group were selected from this resequencing as well as those available in the National Center for Biotechnology Information database of single nucleotide polymorphisms [52] from the nonsequenced areas to be used to select haplotype tagging SNPs. The linkage disequilibrium structure was determined by genotyping SNPs among a reference panel of 349 women from Multi-Ethnic Cohort populations (including 70 Whites). Haplotype frequency estimates were constructed from the genotype data for Whites using the expectation-maximization algorithm to select tag SNPs that maximize prediction of common haplotypes (at *R*^2^_H _≥ 0.7, a measure of correlation between SNPs genotyped and the haplotypes they describe).

We selected six haplotype-tag single nucleotide polymorphisms (htSNPs) that have been identified to capture the genetic variation in the *AR *gene in Caucasians [49] (rs962458, rs6152, rs1204038, rs2361634, rs1337080, and rs1337082), in three haplotype blocks, and considered these as an extended haplotype block [53]. The htSNPs provide a minimum *R*^2^_H _of 0.77 to describe the haplotype diversity among Japanese, Whites, and Latinas from the Multi-Ethnic Cohort selection panel [49]. Genotyping for the *AR *CAG repeat polymorphism was performed as previously described [53]. We selected five htSNPs that have been identified to capture the genetic variation in the *ESR2 *gene in Caucasians [50] (rs3020450, rs1256031, rs1256049, G1730A, and rs944459). The selected htSNPs have a minor allele frequency of 5% or more and *R*^2^_H _>0.7. Three haplotype blocks span the *ESR2 *locus and are highly correlated (*r *>0.95), allowing the analysis of the htSNPs as one block. Based on a report from the Multi-Ethnic Cohort on *CYP19 *haplotype structure and breast cancer risk, we selected 20 SNPs tagging four haplotype blocks, and *R*^2^_H _>0.7 for association with RA risk [40]. These htSNPs were genotyped in HapMap CEU trios to permit an assessment of coverage in relation to the HapMap database (phase II, October 2005) and were estimated to predict 70% of all common SNPs genotyped in the HapMap CEU population across the four linkage disequilibrium blocks. The variants selected for *PGR *were based on functional studies performed by co-investigator IDV [36,54,55] rather than a haplotype tagging method. Nonetheless these SNPs captured 90% of variation in the NHS samples, a predominantly Caucasian population [36,54,55].

### Covariate information

Age was updated in each cycle. Reproductive covariates were chosen based on our past findings of associations between reproductive factors and the risk of developing RA in the NHS [56]. Data on pack-years of smoking (product of years of smoking and packs of cigarettes per day), parity, total duration of breastfeeding (not available in the WHS), age at menarche, menopausal status and postmenopausal hormone use were selected from the questionnaire cycle prior to the date of RA diagnosis (or index date in control individuals).

### Statistical analyses

For analysis of characteristics of cases and controls, we calculated means with standard deviation for continuous variables stratified by cohort. For categorical covariates, we calculated frequencies and percentages. SAS version 9.1.3 software (SAS Institute, Cary, NC, USA) was used for all analyses.

#### Analyses of hormonal factors

We calculated means with standard deviation and medians with range for total testosterone, calculated free testosterone and DHEAS. Threshold values for the quartiles for each hormone were created using the distribution in control individuals. We conducted conditional logistic regression models, conditioned on the matching factors, and adjusted for potential confounders including cigarette smoking and reproductive factors assessed prior to diagnosis of RA for total testosterone, calculated free testosterone and DHEAS, comparing quartiles of continuous hormone levels to estimate relative risks and 95% confidence intervals of RA in the NHS. We repeated the analyses stratified by menopausal status, and by seropositive status.

#### Analyses of gene–hormone associations

Analysis of covariance models were used to evaluate the association of the six *AR *htSNPs and *AR *CAG repeat length with mean plasma hormone levels adjusting for potential confounders, among 89 control samples with both genetic and hormone information in the two NHS blood cohorts. For the total testosterone and calculated free testosterone models, we adjusted for age, body mass index, menopausal status, hormone use and cigarette smoking (never, former, current <15 cigarettes per day and current ≥ 15 cigarettes per day). For DHEAS models, we adjusted for the same covariates as well as the time of day of blood draw.

#### Analyses of genetic factors

We verified Hardy–Weinberg equilibrium for each of the genotypes among control individuals in each of the datasets (NHS blood, NHS cheek cells, NHS II blood, WHS blood). We employed conditional logistic regression analyses, conditioned on matching factors, and adjusted for potential confounders, including cigarette smoking and reproductive factors assessed prior to diagnosis of RA. All analyses were first conducted separately in each cohort and then on data pooled from the three cohorts. As the *P *value for heterogeneity was not significant for the *AR*, *ESR2*, *PGR*, or *CYP19 *genotypes, we also meta-analytically pooled results from the two cohorts using a DerSimonian and Laird random-effects model [57]. In addition, analyses were repeated stratifying by rheumatoid factor status of the RA cases.

We determined common haplotypes in cases and controls using PROC HAPLOTYPE in SAS Genetics. The haplotype dosage estimate was computed using individual genotype data and haplotype frequency estimates from the entire dataset. We pooled rare haplotypes (<5% frequency) into a single category. Conditional logistic regression analyses conditioned on matching factors, and adjusted for potential confounders, were performed for block-specific haplotype associations with RA risk. A likelihood ratio test was performed to test for global haplotype associations, using the most common haplotype as the reference category.

Cochran–Mantel-Haenszel chi-square tests were conducted to evaluate case–control differences in the frequency of the *AR *CAG alleles using multiple cutoff points. In most analyses, we used the cutoff point (CAG)_n _≥ 22 repeats, which has been most frequently used in the literature. We used conditional logistic regression adjusted for potential confounders to assess the increase in log odds of RA per unit increase in CAG repeat, and also examined various cutoff points for repeat length in similar models.

#### Analyses of gene–smoking interactions

We assessed gene–smoking interactions using a multiplicative interaction variable (for example, genotype × smoking) in the conditional logistic regression models. The significance of the interaction was determined using the Wald chi-square test of the interaction variable. In the combined NHS–NHS II nested case–control study dataset, we assessed for interactions between the presence of each polymorphism and cigarette smoking dichotomized as ≤ 10 or >10 pack-years of smoking to account for heavy smoking, as this is the threshold we previously identified to be associated with increased risk of RA [44]. In the WHS cohort we assessed for interaction between polymorphisms and ever/never smoking, as pack-year information was not available.

## Results

Genetic analyses were limited to Caucasian matched pairs to minimize the potential for population stratification. In the NHS cohorts, we confirmed 449 Caucasian RA cases with available DNA and 132 cases with available plasma samples collected prior to first RA symptoms (preclinical RA). In chart reviews, 433 cases had at least four ACR criteria, 14 cases had at least three ACR criteria, and all cases had two rheumatologist reviewers' consensus on RA diagnosis. In the WHS cohort, we confirmed 72 Caucasian RA cases with stored samples. Fifty-eight percent of RA cases in both the NHS and WHS cohorts were rheumatoid factor positive by chart review. In the 132 pre-RA cases with plasma, the mean time between blood draw and RA symptoms was 6.8 years (range = 0 to 14.2 years). The distribution of potential confounders was similar among cases and controls (Table 1). Among RA cases, 179 (67%) were postmenopausal in the NHS cohort and 39 (54%) were postmenopausal in the WHS cohort at blood draw.

**Table 1:** Characteristics of rheumatoid arthritis cases and matched controls in the NHS and WHS

	NHS (449 cases/449 controls)		WHS (72 cases/202 controls)	
		
	RA cases	Controls	RA cases	Controls
Matching factors				
Age at blood draw	55 ± 8	55 ± 8	55 ± 8	55 ± 8
Postmenopausal^a^	179 (67%)	179 (67%)	39 (54%)	109 (54%)
Current postmenopausal hormone use	92 (34%)	93 (35%)	37 (51%)	104 (51%)
Other characteristics				
Ever cigarette smokers	272 (61%)	252 (56%)	46 (64%)	106 (52%)
Pack-years among smokers	23 ± 16	23 ± 20	-	-
Parous	411 (92%)	420 (94%)	62 (86%)	180 (89%)
Breastfed babies ≥ 12 months total^b^	67 (15%)	81 (18%)	-	-
Age at menarche <12 years	107 (24%)	96 (22%)	18 (25%)	47 (23%)
Irregular menstrual cycles	66 (15%)	61 (14%)	-	-
Body mass index (kg/m^2^)	26 ± 5	26 ± 5	26 ± 6	26 ± 5
Rheumatoid arthritis characteristics				
Age at diagnosis	55 ± 10		61 ± 8	
Rheumatoid factor positive	260 (58%)		42 (58%)	
Cyclic citrullinated antibody positive^c^	73 (55%)		-	
Rheumatoid nodules	59 (13%)		9 (13%)	
Radiographic changes	137 (31%)		15 (21%)	
Hormone levels^d^				
Free testosterone (pg/ml)	0.06 (0.03)	0.06 (0.03)		
Free testosterone (pg/ml)	0.06 (0.05 to 0.08)	0.06 (0.04 to 0.08)		
Total testosterone (pg/ml)	22.0 ± 10.0	21.7 ± 11.1		
Total testosterone (pg/ml)	22.0 (16.0 to 31.0)	21.0 (16.0 to 29.0)		
DHEAS (pg/ml)	91.1 ± 68.9	87.5 ± 59.0		
DHEAS (pg/ml)	72.1 (38.5 to 113.9)	69.0 (46.0 to 116.4)		

Data presented as mean ± standard deviation, *n *(%) or median (25th to 75th percentile). ^a^Percentage is calculated among postmenopausal women or parous women, with unknown/missing group excluded. For the rest of the variables, percentage was calculated with missing category included. ^b^Calculated among parous women in the Nurses' Health Study (NHS), data not available in the Women's Health Study (WHS). ^c^Cyclic citrullinated peptide antibodies tested in 132 preclinical rheumatoid arthritis (RA) samples. ^d^Measured in NHS samples for free and total testosterone and dehydroepiandrosterone sulfate (DHEAS) (n = 132 rheumatoid arthritis cases, n = 396 controls).

### Plasma androgens in cases compared with controls in the NHS

Comparing the lowest quartile with the highest quartile of total testosterone or calculated free testosterone there were no significant associations with RA risk in univariate analysis or after adjusting for confounders (Table 2). For example, the top quartile of calculated free testosterone level was associated with a relative risk of 1.7 (95% confidence interval = 0.8 to 3.5). Similarly for DHEAS, there was no evidence of increased RA risk with low DHEAS in either univariate or multivariable analysis. Stratified analyses among premenopausal women and postmenopausal women and among seropositive or seronegative RA had similar results (data not shown).

**Table 2:** Rheumatoid arthritis associated with quartiles of plasma androgens in the Nurses' Health Studies

	Quartile 1	Quartile 2	Quartile 3	Quartile 4	*P *trend^a^	*P *continuous^b^
Free testosterone						
Median (controls)	0.028	0.046	0.061	0.085		
Cases/controls	25/98	36/98	37/98	33/98		
Unadjusted^c^	1.0	1.6 (0.8 to 2.9)	1.7 (0.9 to 3.2)	1.5 (0.8 to 3.0)	0.36	0.51
Adjusted^d^	1.0	1.5 (0.8 to 2.8)	1.6 (0.8 to 3.3)	1.7 (0.8 to 3.5)	0.24	0.44
Total testosterone						
Median (controls)	11.0	18.0	23.0	34.0		
Cases/controls	32/104	33/103	31/96	36/92		
Unadjusted^c^	1.0	1.1 (0.6 to 1.9)	1.1 (0.6 to 2.1)	1.4 (0.7 to 2.5)	0.32	0.75
Adjusted^d^	1.0	1.1 (0.6 to 2.0)	1.2 (0.6 to 2.4)	1.5 (0.8 to 2.9)	0.20	0.52
Dehydroepiandrosterone sulfate						
Median (controls)	30.58	56.16	93.10	158.20		
Cases/controls	40/98	20/98	37/98	34/98		
Unadjusted^c^	1.0	0.5 (0.3 to 0.9)	0.9 (0.5 to 1.6)	0.8 (0.5 to 1.5)	0.85	0.51
Adjusted^d^	1.0	0.5 (0.3 to 1.0)	0.9 (0.5 to 1.5)	0.7 (0.4 to 1.4)	0.66	0.71

Data presented as relative risk (95% confidence interval), n = 132 preclinical rheumatoid arthritis cases/396 controls. ^a^Calculated using median hormone level in each quartile and the Wald chi-square test. ^b^Calculated using continuous hormone levels and the Wald chi-square test. ^c^Conditional logistic regression conditioned on strata defined by matched factors. ^d^Conditional logistic regression conditioned on matching factors, adjusted for body mass index (continuous), cigarette smoking (never, past, current smoker <15 cigarettes per day, current smoker ≥ 15 cigarettes per day), age at menarche, menstrual regularity, parity, breastfeeding.

### Androgen receptor polymorphisms and hormone levels in the NHS

There were modest associations between several *AR *SNPs and plasma hormone levels among control samples (Table 3). The variant genotypes for rs962458, rs6152, rs1204038 and rs1337080 were associated with higher plasma calculated free testosterone levels (*P *= 0.03, *P *= 0.03, *P *= 0.05 and *P *= 0.03, respectively) and rs6152 was associated with total testosterone (*P *= 0.04). We found no significant association between the length of the *AR *CAG repeat and hormone levels among 89 control samples (data not shown).

**Table 3:** Association of *AR *haplotype-tag polymorphisms and plasma hormone levels in the Nurses' Health Studies

*AR *SNP	Dehydroepiandrosterone sulfate^a^	Total testosterone^b^	Free testosterone^c^
rs962458			
CC	96.6 ± 12.6	23.2 ± 1.4	0.062 ± 0.003
CT/TT	98.5 ± 19.2	30.2 ± 3.4	0.081 ± 0.008
*P *value	0.91	0.06	0.03
rs6152			
AA	84.7 ± 14.0	22.5 ± 1.5	0.060 ± 0.004
AG/GG	109.8 ± 14.4	28.8 ± 2.6	0.076 ± 0.006
*P *value	0.07	0.04	0.03
rs1204038			
TT	86.7 ± 14.3	22.1 ± 1.5	0.059 ± 0.004
TC/CC	104.9 ± 13.8	27.4 ± 2.2	0.073 ± 0.006
*P *value	0.17	0.06	0.05
rs2361634			
GG	97.1 ± 12.5	24.2 ± 1.4	0.064 ± 0.003
GA/AA	92.1 ± 19.1	24.3 ± 3.4	0.067 ± 0.008
*P *value	0.76	0.96	0.71
rs1337080			
GG	96.4 ± 12.5	23.2 ± 1.4	0.061 ± 0.003
GA/AA	98.6 ± 19.1	30.2 ± 3.4	0.080 ± 0.008
*P *value	0.89	0.06	0.03
rs1337082			
GG	87.1 ± 14.4	22.7 ± 1.8	0.060 ± 0.004
GA/AA	101.8 ± 13.2	25.4 ± 1.9	0.068 ± 0.005
*P *value	0.22	0.32	0.20

Androgen receptor gene (*AR*) association in 89 controls from the Nurses' Health Studies. Data presented as mean ± standard deviation. ^a^Analysis of covariance adjusted for age (continuous), body mass index (continuous), menopausal status and postmenopausal hormone use, cigarette smoking (never, past, current smoker <15 cigarettes per day, current smoker ≥ 15 cigarettes per day) and time of day of blood draw. ^b^Analysis of covariance adjusted for age (continuous), body mass index (continuous), menopausal status and postmenopausal hormone use, and cigarette smoking (never, past, current smoker <15 cigarettes per day, current smoker ≥ 15 cigarettes per day). ^c^Analysis of covariance adjusted for age (continuous), body mass index (continuous), menopausal status and postmenopausal hormone use, and cigarette smoking (never, past, current smoker <15 cigarettes per day, current smoker ≥ 15 cigarettes per day).

### Androgen receptor polymorphisms in the NHS and the WHS

Using a dominant model there was no association with RA for any of the six htSNPs in the NHS, in the WHS, and in the pooled sample with 522 cases and 662 controls in adjusted analyses (see Table S1 in Additional data file 1). In pooled analyses, with stratification into rheumatoid factor-positive and rheumatoid factor-negative RA, the odds ratios were modestly elevated for rheumatoid factor-positive RA but remained nonsignificant (see Table S2 in Additional data file 1). Haplotype analysis had similar null results (Table 4). There was no evidence for any interaction between the six htSNPs in the androgen receptor and cigarette smoking classified as never/ever smoked in the combined analysis, or classified as never/≤ 10 pack-years of smoking versus >10 pack-years of smoking in the NHS analysis (data not shown). We found no significant association between the length of the *AR *CAG repeat and RA risk (data not shown).

**Table 4:** Associations of *AR *haplotypes, *ESR2 *haplotypes, *PGR *haplotypes, *CYP19*, block 1 haplotypes and RA risk

	Cases (%)	Controls (%)	Odds ratio (95% confidence interval)^a^	Odds ratio (95% confidence interval)^b^
*AR *long-range haplotypes^c^				
0-0-0-0-0-0	71.2	69.7	1.00 reference	1.00 reference
0-1-1-0-0-1	8.1	8.1	0.98 (0.72 to 1.33)	0.99 (0.72 to 1.36)
0-0-0-1-0-0	6.7	8.0	0.86 (0.62 to 1.19)	0.82 (0.58 to 1.14)
1-1-1-0-1-1	6.4	5.3	1.19 (0.82 to 1.71)	1.29 (0.88 to 1.88)
0-0-0-0-0-1	4.7	6.1	0.73 (0.50 to 1.07)	0.76 (0.51 to 1.12)
*ESR2 *haplotypes^d^				
0-1-0-0-0	42.4	46.8	1.00	1.00
1-0-0-1-0	27.2	24.7	1.16 (0.95 to 1.42)	1.15 (0.94 to 1.41)
0-0-0-0-0	10.9	8.3	1.47 (1.07 to 2.01)	1.44 (1.05 to 1.98)
0-0-0-1-0	9.1	9.6	0.99 (0.71 to 1.38)	0.91 (0.65 to 1.28)
*PGR *haplotypes^e^				
0-0-0-0	41.2	37.5	1.00	1.00
0-0-1-0	34.4	34.2	0.93 (0.75 to 1.14)	0.92 (0.75 to 1.14)
0-0-0-1	13.1	16.3	0.72 (0.55 to 0.94)	0.73 (0.56 to 0.95)
1-0-0-0	6.3	7.4	0.75 (0.53 to 1.06)	0.75 (0.53 to 1.07)
*CYP19*, block 1 haplotypes^f^				
0-0-1-0-0-0	39.2	31.2	1.00	1.00
0-0-0-1-1-0	14.0	14.4	0.68 (0.50 to 0.94)	0.71 (0.52 to 0.98)
0-0-0-0-1-0	10.4	14.4	0.81 (0.61 to 1.08)	0.83 (0.62 to 1.11)
0-0-0-0-0-0	10.3	9.6	0.69 (0.51 to 0.93)	0.71 (0.52 to 0.96)
1-1-0-0-1-1	5.5	6.5	0.58 (0.38 to 0.88)	0.58 (0.38 to 0.88)

RA, rheumatoid arthritis. ^a^Conditional logistic regression, conditioning on matching factors. ^b^Conditional logistic regression, conditioning on matching factors and adjusting for cigarette smoking (never, past, current smoker <15 cigarettes per day, current smoker ≥ 15 cigarettes per day), age at menarche and parity. ^c^Androgen receptor gene (*AR*) haplotype-tagged SNPs: rs962458-rs6152-rs1204038-rs2361634-rs1337080-rs1337082. ^d^Estrogen receptor 2 gene (*ESR2*) haplotype-tagged SNPs: rs3020450-rs1256031-rs1256049-rs4986938-rs944459. ^e^Progesterone receptor gene (*PGR*) haplotype-tagged SNPs: rs518162-rs10895068-rs1379130-rs1042839. ^f^Aromatase gene (*CYP19*) block 1 haplotype-tagged SNPs: rs2446405-rs2445765-rs2470144-rs2445762-rs1004984-rs1902584; global haplotype association, *P *= 0.02.

### *ESR2*, *PGR*, and *CYP19* polymorphisms in the NHS and the WHS

For *ESR2*, *PGR *and *CYP19*, none of the individual htSNPs were significantly associated with RA. A single haplotype in *ESR2 *and a single haplotype in *PGR *were associated with RA; however, the global tests for haplotype association were not significant for *ESR2 *(*P *= 0.06) and for *PGR *(*P *= 0.21) (Table 4). There was no association for haplotypes in *CYP19*, blocks 2, 3, or 4 (data not shown). There were four haplotypes in *CYP19*, block 1 that were modestly associated with RA in adjusted analyses (Table 4), with a significant global test for haplotype association (*P *= 0.02). None of these findings were significant after adjustment for multiple comparisons.

## Discussion

We found no evidence that women with pre-RA have lower plasma androgen levels than matched control individuals in this nested case–control study that included 132 incident RA cases with plasma blood samples collected prior to disease onset and 396 controls. This finding is consistent with a case–control study nested in a Finnish cohort of 19,072 subjects, which demonstrated no differences in concentration of total testosterone and DHEAS measured in stored serum specimens collected up to 16 years prior to diagnosis between 116 pre-RA cases (32 men, 84 women) and controls [18]. Serum SHBG was not measured in that study, and therefore the biologically active form – free testosterone – could not be determined. In contrast, a smaller study demonstrated low DHEAS among 11 premenopausal pre-RA women compared with control individuals, with levels measured 7 to 18 years prior to RA onset. In stratified analyses, however, we could not confirm an association between DHEAS and RA in premenopausal women. The observed androgen deficiency reported in the literature in existing RA [6-10] is most probably a consequence of the disease, and not causal.

In a small sample of 89 controls with both genotype and plasma hormone results, we demonstrated that four of the *AR *SNPs were associated with higher free testosterone levels – suggesting that these polymorphisms may have functional effects, although the sample size is small. We found no evidence, however, of polymorphisms in the *AR *gene being associated with RA risk in unadjusted analyses or after adjustment for potential confounders. Similarly, none of the individual polymorphisms in *ESR2*, *PGR*, and *CYP19 *or in the *AR *CAG repeat was associated with RA risk. There was some modest evidence for haplotype associations in *ESR2*, *PGR *and *CYP19*, block 1; however, none of these results were significant after adjustment for multiple comparisons.

Studies regarding repeat polymorphisms in *AR *suggest associations between CAG repeat length and clinical features of RA but no association with RA susceptibility [58-60]. Associations regarding *ESR2 *repeat polymorphisms and clinical features of RA have been reported [61,62]. An association between *CYP19 *and RA was reported in a linkage study [63]. No case–control association studies for SNPs or haplotypes, however, have been reported for *AR*, *ESR2*, *PGR*, and *CYP19*. None of the published genome-wide association studies have reported significant findings for these genes either [64,65].

The prospective design of these cohorts allowed us to study plasma androgen levels up to 14 years prior to RA onset and to adjust for a number of potential confounders such as cigarette smoking and reproductive factors. There were several limitations to the study design, however, including the small number of incident RA cases after the blood collection, which limited the power to detect hormone associations, and the lack of repeated blood samples at multiple timepoints prior to RA. Data from the NHS, however, indicate that a single sample reflects long-term hormone levels reasonably well. For instance, postmenopausal hormone levels measured three times over a 3-year period in 79 women from the NHS reliably categorized average levels with intraclass correlations of 0.88 for testosterone, 0.88 for DHEAS, and 0.92 for SHBG [66]. We studied plasma androgens rather than local androgen levels such as synovial fluid levels that may be more indicative of an altered estrogen–androgen balance in the pathophysiology of RA [14]. With 132 cases and 396 controls we have 80% power to detect an odds ratio of 0.38 for the top quartile as compared with the lowest quartile. For the genetic analyses, we limited the analysis to women with self-reported Caucasian ancestry to minimize population stratification, and other studies have reported little stratification in this cohort [67]. With 521 cases and 651 controls, we had 86% power to detect an odds ratio of 1.5 for minor allele frequencies of 15%, but only 27% power to detect odds ratios of 1.2. The power to detect modest odds ratios, such as those demonstrated in recent genome-wide association studies in RA [68-70], was therefore quite limited.

## Conclusions

Although the possibility of a biologic relationship between *AR*, androgen levels, and RA risk is intriguing, our findings do not suggest that *AR *is related to RA risk in women. We do not show any significant associations for other hormone-related genes, *ESR2*, *PGR *and *CYP19 *and RA risk after adjustment for multiple comparisons. Steroid hormone levels measured at a single timepoint from 0 to 14 years prior to RA onset were not associated with RA risk in the present study. In conclusion, among women in the NHS, NHS II and WHS, neither hormone receptor genes nor plasma steroid hormone levels are associated with RA risk.

## Abbreviations

ACR: American College of Rheumatology; *AR*: androgen receptor gene; *CYP19*: aromatase gene; DHEAS: dehydroepiandrosterone sulfate; *ESR2*: estrogen receptor 2 gene; htSNP: haplotype-tagged single nucleotide polymorphism; IL: interleukin; NHS: Nurses' Health Study; *PGR*: progesterone receptor gene; RA: rheumatoid arthritis; SHBG: sex hormone binding globulin; SNP: single nucleotide polymorphism; TNF: tumor necrosis factor; WHS: Women's Health Study.

## Competing interests

PAF receives salary from Genzyme Corporation. Genzyme Corporation will not in any way gain or lose financially from the publication of the present manuscript, either now or in the future. Genzyme is not financing this manuscript. PAF holds Genzyme Corporation stocks and shares that will not in any way gain or lose financially from the publication of this manuscript, either now or in the future.

## Authors' contributions

EWK participated in the study design, data acquisition, analysis and interpretation of data, and manuscript preparation. LBC participated in the study design, statistical analysis and interpretation of data, and manuscript preparation. MM participated in the study design, statistical analysis and interpretation of the data. S-CC participated in statistical analysis. BTK participated in statistical analysis and manuscript preparation. KHC participated in data acquisition, interpretation of data, and manuscript preparation. PAF participated in the study design and interpretation of the data. ST participated in the study design, statistical analysis and interpretation of data, and manuscript preparation. SEH participated in interpretation of the data and manuscript preparation. I-ML participated in the study design and interpretation of the data. JB participated in the study design and interpretation of the data. IDV participated in the study design, interpretation of the data, and manuscript preparation.

## Supplementary Material

Additional file 1 A Word file containing two tables that list the association of htSNPs in the *AR *gene and RA. Table S1 presents the association of the six htSNPs in the *AR *gene with RA in the NHS, in the WHS, and in the pooled sample. Table S2 presents the association of the six htSNPs in the *AR *gene with seropositive RA and seronegative RA in the NHS, in the WHS, and in the pooled sample. Click here for file
