# Supplementary material for: A prospective study of androgen levels, hormone-related genes and risk of rheumatoid arthritis
Source: Arthritis Res Ther. 2009 Jun 25;11(3):R97. doi: 10.1186/ar2742 (PMC2714153; doi:10.1186/ar2742)
Supplement: Additional file 1 — A Word file containing two tables that list the association of htSNPs in the AR gene and RA. Table S1 presents the association of the six htSNPs in the AR gene with RA in the NHS, in the WHS, and in the pooled sample. Table S2 presents the association of the six htSNPs in the AR gene with seropositive RA and seronegative RA in the NHS, in the WHS, and in the pooled sample. [file ar2742-S1.doc]

| **Supplementary Table 1. Association of AR haplotype-tag polymorphisms and the risk of RA in Nurses’ Health Studies and Women’s Health Study, N=521 caes, 651 controls** | | | | | | | | | | | | |
| --- | --- | --- | --- | --- | --- | --- | --- | --- | --- | --- | --- | --- |
|  |  |  | **NHS^1^** | | | | **WHS^2^** | | | | **Meta^3^** | |
| **SNP** | **Major Allele** | **Minor Allele** | **MAF Cases** | **MAF Controls** | **Dominant**  **OR (95% CI)** | **p-value** | **MAF of Cases** | **MAF of Controls** | **Dominant**  **OR (95% CI)** | **p-value** | **Dominant**  **OR (95% CI)** | **p-value** |
| rs962458 | C | T | 0.07 | 0.06 | 1.38 (0.87-2.18) | 0.17 | 0.08 | 0.06 | 1.38 (0.62-3.07) | 0.43 | 1.38 (0.93-2.05) | 0.11 |
| rs6152 | A | G | 0.16 | 0.15 | 1.11 (0.82-1.50) | 0.50 | 0.18 | 0.14 | 1.33 (0.70-2.50) | 0.38 | 1.15 (0.87-1.51) | 0.32 |
| rs1204038 | T | C | 0.16 | 0.16 | 1.07 (0.79-1.44) | 0.65 | 0.20 | 0.15 | 1.30 (0.72-2.38) | 0.39 | 1.11 (0.85-1.45) | 0.43 |
| rs2361634 | G | A | 0.06 | 0.08 | 0.68 (0.45-1.02) | 0.06 | 0.09 | 0.09 | 1.21 (0.55-2.69) | 0.63 | 0.82 (0.48-1.39) | 0.46 |
| rs1337080 | G | A | 0.07 | 0.06 | 1.30 (0.84-2.01) | 0.23 | 0.09 | 0.06 | 1.91 (0.85-4.25) | 0.12 | 1.42 (0.97-2.08) | 0.07 |
| rs1337082 | G | A | 0.19 | 0.21 | 0.97 (0.73-1.28) | 0.81 | 0.21 | 0.20 | 1.03 (0.57-1.86) | 0.92 | 0.98 (0.76-1.26) | 0.86 |

^1^NHS: Conditional logistic regression adjusting for, age at menarche, parity, breast feeding, and cigarette smoking (never, past, current smoker <15 cigarettes per day, current smoker ­≥15 cigarettes per day), n = 449 cases, 449 controls

^2^ WHS: Conditional logistic regression adjusting for cigarette smoking (never, past, current smoker <15 cigarettes per day, current smoker ­≥15 cigarettes per day), age at menarche and parity n = 72 cases, 202 controls

^3^ Pooled NHS/WHS: Conditional logistic regression adjusting for cigarette smoking (never, past, current smoker <15 cigarettes per day, current smoker ­≥15 cigarettes per day), age at menarche and parity, n = 521 cases, 651 controls

^1^Unconditional logistic regression adjusting for cigarette smoking (never, past, current smoker <15 cigarettes per day, current smoker ­≥15 cigarettes per day),

year of birth, age at menarche and parity

| **Supplementary Table 2. Association of AR haplotype-tag polymorphisms and the risk of RA in NHS, NHSII and WHS stratified by rheumatoid factor status** | | | | | | | | | | |
| --- | --- | --- | --- | --- | --- | --- | --- | --- | --- | --- |
|  |  |  | **302 RF+ Cases/651 Controls^1^** | | | | **219 RF- Cases/651 Controls^1^** | | | |
| **SNP** | **Major Allele** | **Minor Allele** | **MAF of Cases** | **MAF of Controls** | **Dominant**  **OR (95% CI)** | **p-value** | **MAF of Cases** | **MAF of Controls** | **Dominant**  **OR (95% CI)** | **p-value** |
| rs962458 | C | T | 0.08 | 0.06 | 1.45 (0.97-2.19) | 0.07 | 0.06 | 0.06 | 1.01 (0.61-1.65) | 0.98 |
| rs6152 | A | G | 0.17 | 0.15 | 1.33 (0.97-1.81) | 0.08 | 0.14 | 0.15 | 0.92 (0.64-1.32) | 0.66 |
| rs1204038 | T | C | 0.18 | 0.15 | 1.31 (0.96-1.78) | 0.09 | 0.14 | 0.15 | 0.86 (0.60-1.23) | 0.40 |
| rs2361634 | G | A | 0.06 | 0.08 | 0.81 (0.53-1.22) | 0.31 | 0.07 | 0.08 | 0.80 (0.51-1.27) | 0.35 |
| rs1337080 | G | A | 0.08 | 0.06 | 1.48 (0.98-2.25) | 0.06 | 0.06 | 0.06 | 1.05 (0.64-1.73) | 0.84 |
| rs1337082 | G | A | 0.21 | 0.20 | 1.08 (0.80-1.45) | 0.61 | 0.18 | 0.20 | 0.85 (0.61-1.18) | 0.32 |
